# SARS-CoV-2 Infections and Impact of the COVID-19 Pandemic in Pregnancy and Breastfeeding: Results from an Observational Study in Primary Care in Belgium

**DOI:** 10.3390/ijerph17186766

**Published:** 2020-09-17

**Authors:** Michael Ceulemans, Jan Y. Verbakel, Kristel Van Calsteren, An Eerdekens, Karel Allegaert, Veerle Foulon

**Affiliations:** 1 Clinical Pharmacology and Pharmacotherapy, Department of Pharmaceutical and Pharmacological Sciences, KU Leuven, Herestraat 49, 3000 Leuven, Belgium; karel.allegaert@kuleuven.be (K.A.); veerle.foulon@kuleuven.be (V.F.); 2 Academic Center for General Practice, Department of Public Health and Primary Care, KU Leuven, Kapucijnenvoer 33, 3000 Leuven, Belgium; jan.verbakel@kuleuven.be; 3 Department of Obstetrics and Gynecology, University Hospitals Leuven Gasthuisberg, Herestraat 49, 3000 Leuven, Belgium; kristel.vancalsteren@uzleuven.be; 4 Woman and Child, Department of Development and Regeneration, KU Leuven, Herestraat 49, 3000 Leuven, Belgium; an.eerdekens@uzleuven.be; 5 Department of Pediatrics and Neonatology, University Hospitals Leuven Gasthuisberg, Herestraat 49, 3000 Leuven, Belgium; 6 Department of Clinical Pharmacy, Erasmus MC Sophia Children’s Hospital, Zimmermanweg 11, 3015 CP Rotterdam, The Netherlands

**Keywords:** COVID-19, SARS-CoV-2, pregnancy, breastfeeding, counseling, social support, community health services, public health, primary health care, Belgium

## Abstract

COVID-19 also affects pregnant and breastfeeding women. Hence, clinicians and policymakers require reliable evidence on COVID-19 epidemiology and consequences in this population. We aimed to assess the susceptibility of pregnant women to SARS-CoV-2 and women’s perceived impact of the pandemic on their breastfeeding practices, medical counseling and social support. We performed a cross-sectional study using an online survey in primary care in Belgium. Pregnant and breastfeeding women and women who breastfed in the preceding four weeks were eligible to participate. The survey was distributed through social media in April 2020. In total, 6470 women participated (i.e., 2647 pregnant and 3823 breastfeeding women). Overall, 0.3% of all respondents reported to have tested positive for SARS-CoV-2, not indicating a higher susceptibility of pregnant women to contracting COVID-19. More than 90% refuted that the pandemic affected their breastfeeding practices, nor indicated that the coronavirus was responsible for breastfeeding cessation. Half of the women even considered giving longer breastmilk because of the coronavirus. In contrast, women’s medical counseling and social support were negatively affected by the lockdown. Women without previous breastfeeding experience and in the early postpartum period experienced a higher burden in terms of reduced medical counseling and support. In the future, more consideration and alternative supportive measures such as tele-visits by midwives or perinatal organizations are required for these women.

## 1. Introduction

In December 2019, the first cases of COVID-19 were identified in Wuhan, China [1]. On 11th March 2020, the World Health Organization declared this global health emergency as a pandemic [2]. In Belgium, the first case of COVID-19 was identified on 3rd February 2020. Due to the exponential increase in the number of affected people, hospitalizations and deaths, the Belgian government imposed strict containment and social isolation measures (i.e., lockdown) from 18th March 2020 onwards. 

Similar to other populations, pregnant and breastfeeding women encounter SARS-CoV-2 and might contract COVID-19 [3]. However, given the unique situation of pregnancy and breastfeeding, perinatal COVID-19 entails the risk of affecting the (unborn) infant [4]. Unfortunately, due to the novelty of SARS-CoV-2, little is known about the susceptibility of pregnant women to the virus [5,6]. Nevertheless, this information is critical, particularly as the previous coronaviruses SARS and MERS have been associated with severe fetal–maternal complications [7,8]. From a theoretical perspective, pregnant women are more susceptible to respiratory pathogens and the development of severe pneumonia due to the changes in their immune system [9]. So far, mainly case-reports and case-series have been published concerning COVID-19 in the perinatal period [3,10,11,12,13], preventing the calculation of risk estimates. In addition, most studies have focused on hospitalized patients, although a maximum of one in five of COVID-19 patients in the general population require hospital admission [14]. Hence, limiting research initiatives to hospital settings leads to an underuse of the research potential of primary care. 

Besides the potential impact of COVID-19 on fetal–maternal health, the emergence of a new and highly contagious virus, including the unprecedented containment and social isolation measures, might have created (exceptional) challenges for perinatal and child healthcare [15]. In Belgium, it is common practice for pregnant women to consult with several types of health care professionals (HCPs) throughout their pregnancy (i.e., obstetrician, midwife, general practitioner (GP) or medical specialist). In the postpartum period, additional HCPs such as pediatricians or lactation consultants and perinatal organizations play a role in the medical follow-up of the mother and child, help with breastfeeding, taking care of the newborn and household support. To date, it is unknown to what extent women’s access to health services and medical counseling has been affected by the imposed restrictions in Belgium. 

Overall, the challenges HCPs and policymakers face due to the pandemic require prompt solutions. From an epidemiological perspective, insight into how many pregnant and breastfeeding women have been infected with SARS-CoV-2 is required. At least as relevant, from a public health perspective, evidence on the impact of the pandemic on breastfeeding practices, medical care and social support is vital, including whether personal pregnancy and breastfeeding experiences are associated with women’s self-reported burden. Understanding the consequences of the pandemic on fetal–maternal health will not only contribute to reducing adverse events, but will also allow for the optimization of the organization of perinatal healthcare during subsequent waves. 

Therefore, the current study, performed in Belgian primary care, aimed to provide estimates of SARS-CoV-2 infections among pregnant and breastfeeding women, as well as to assess women’s perceived impact of the pandemic on their breastfeeding practices, medical counseling and social support during pregnancy and lactation.

## 2. Methods

### 2.1. Study Design and Sample

A cross-sectional, observational study using an online, anonymous survey was conducted among pregnant and breastfeeding women living in Belgium. The questionnaire was distributed in April 2020, at least four weeks after the start of the lockdown in Belgium. All pregnant and breastfeeding women and women who breastfed in the four weeks prior to the survey, older than 18 years, and understanding Dutch or French were eligible to participate. A Dutch and French version of the survey was hosted on an online survey platform (i.e., Qualtrics) which participants could access by using a URL or QR code. Ethical approval of the local Ethics Committee Research UZ/KU Leuven (S63966; 10 April 2020) and online informed consent of all participants were obtained. 

### 2.2. Survey

The survey was part of a large COVID-19 research project performed in Belgium and aimed at promptly studying (1) the extent of SARS-CoV-2 infections among pregnant and breastfeeding women; (2) women’s information needs about the coronavirus; (3) the impact of the pandemic and lockdown on perinatal mental health, medication use and perceptions, breastfeeding practices, medical counseling, and social support. The results on breastfeeding practices, medical counseling and social support, along with COVID-19 epidemiology, are the subject of this paper. The surveys for pregnant and breastfeeding women were quite similar, except for the questions on breastfeeding practices, and were exploratory. In both surveys, COVID-19 epidemiology was studied by asking questions which SARS-CoV-2 tests, test results and symptoms women had experienced in the preceding four weeks. A pilot version of both surveys was tested by three women each and amendments were made accordingly. Survey translation into French was performed by a professional translation office. 

With regard to survey promotion, the URL, QR code, and posters to invite women were distributed via the websites and social media accounts of perinatal organizations, patient and advocacy groups, professional organizations of HCPs, as well as via popular pregnancy and breastfeeding fora and Facebook groups. The Dutch version of the survey was available online between 10–19 April 2020; the French version could be completed between 25 April and 3 May 2020. 

### 2.3. Data Analysis

Survey results were analyzed using descriptive statistics with SPSS Statistics version 26 (IBM Corp, Armonk, NY, USA ). All women who completed the breastfeeding survey, including those who recently quit breastfeeding, were grouped into the category “breastfeeding women”. The breastfeeding duration, as reported at the time of survey completion, was categorized into “≤6 weeks”, “between 6 weeks and 6 months”, and “>6 months”. Any formal degree obtained after a secondary school was considered as “higher education”. With respect to COVID-19 diagnosis, women were categorized as COVID-19 case if they reported having received laboratory confirmation of SARS-CoV-2 infection, irrespective of clinical signs and symptoms. Chi-square and Fisher’s exact tests (the latter in case requirements for chi-square tests were not fulfilled) were used to determine whether personal pregnancy and breastfeeding experiences (i.e., a previous pregnancy or breastfeeding experience and the categorized duration of the current breastfeeding period) are associated with the self-reported impact of the pandemic on medical counseling and social support. A 5% significance level was assumed for all tests. 

## 3. Results

### 3.1. Characteristics of Study Participants 

In total, 6470 women participated in the survey (2647 pregnant and 3823 breastfeeding women). The mean age of the pregnant and breastfeeding women was 31 ± 4 years and 32 ± 4 years, respectively. In total, 82% were highly educated, and 92% were professionally active (40% in healthcare). Primi- and multigravida were equally represented among the pregnant respondents, while 12% were in the first gestational trimester, 43% in the second trimester, and 45% in the third trimester. Of the breastfeeding women, 54% already had previous breastfeeding experience. Overall, less than 10% of all respondents reported to be involved in another COVID-19 research project. A detailed overview of the study participants’ characteristics is shown in the Supplementary Materials (Table S1).

### 3.2. SARS-CoV-2 Infections in the Study Sample

In total, only a few pregnant (3%) and breastfeeding women (4%) reported that they had been tested for SARS-CoV-2 infection. The test positivity rate among pregnant and breastfeeding women was 13% (*n* = 9) and 6% (*n* = 8), respectively (see Table 1). This corresponds to 0.3% of all study respondents who tested positive for SARS-CoV-2. Only 1 of the 17 COVID-19 cases had been hospitalized. In addition, 0.5% of all respondents reported they were living together with someone who had tested positive for SARS-CoV-2 in the preceding four weeks. 

### 3.3. Self-Reported Symptoms of the COVID-19 Cases 

The 17 COVID-19 cases mainly suffered from headache (*n* = 13), fever (*n* = 12), anosmia/dysgeusia/anorexia (*n* = 11), and muscle pain (*n* = 11). Cough (*n* = 9) and shortness of breath (*n* = 8) were also often reported. Overall, eight of the nine pregnant women with COVID-19 reported that the symptoms had a (rather) large influence on their functioning, while this was the case for four of the eight breastfeeding women. A detailed overview of the self-reported symptoms of the pregnant and breastfeeding COVID-19 cases is included in the Supplementary Materials (Table S2). 

### 3.4. Impact on Breastfeeding Practices

Overall, 97% of the breastfeeding women were still breastfeeding at the time of survey completion. Most breastfed infants were >6 months (42%) or between 6 weeks and 6 months (38%). Only one-fifth of infants was ≤6 weeks (20%). Of the breastfed infants, 53% currently received mother’s milk exclusively. 

In total, 91% of the breastfeeding women reported that the infant’s diet had not changed due to the coronavirus pandemic. In case the diet had changed since the coronavirus outbreak, 82% cited that their infants received more often breastmilk compared to the period preceding the pandemic. The main reasons for this increase were that staying home with the infant (as a result of the lockdown) facilitated breastfeeding, and the woman’s desire to protect her infant against coronavirus through breastmilk. The main reasons for a decline in breastfeeding, as reported by the women, were a reduction in milk production due to concerns about the virus, and the combination with other childcare responsibilities at home. 

Moreover, 88% of the few women who stopped breastfeeding in the four weeks prior to the survey indicated that breastfeeding cessation was not caused by the coronavirus. In case the coronavirus was considered responsible for breastfeeding cessation, women mainly attributed it to the consequences of the lockdown (i.e., work from home along with other childcare responsibilities), a higher workload, or reduced milk production due to concerns about the virus. In contrast, 97% of the breastfeeding women had not considered (at all) to stop giving breastmilk due to the coronavirus. In fact, 55% had already (strongly) considered as a result of the coronavirus to give breastmilk for a longer period of time. Finally, 86% of the women with previous breastfeeding experience felt that the coronavirus had (rather) no influence on how they personally dealt with breastfeeding.

### 3.5. Impact on Medical Counseling during Pregnancy and Lactation 

In total, 86% of all pregnant respondents answered that their pregnancy was mainly followed-up by an obstetrician, while a midwife and a GP were involved as the main HCP in the medical follow-up of 11% and 3% of the pregnant women, respectively. Importantly, 53% of the pregnant women indicated that the coronavirus pandemic influenced their current pregnancy follow-up to some extent. Of those, at least 60% reported having received less follow-up by midwives (65%) and obstetricians (62%) compared to before the pandemic (see Table 2). Likewise, more than 40% cited that the pandemic negatively influenced the extent of medical counseling by medical specialists (42%) and GPs (42%). In contrast, less than 10% mentioned having received more follow-up by any type of HCP. 

Furthermore, 43% of the breastfeeding women reported having experienced some impact of the pandemic on the extent of medical counseling during the breastfeeding period. Of those, more than 50% answered to have received less counseling by any type of HCPs compared to before the pandemic (see Table 2). In fact, the counseling by the official perinatal organizations focusing on the wellbeing of newborns and breastfed infants was even more negatively affected (84%). In contrast, less than 10% mentioned having received more follow-up by any type of HCP. 

With regard to women’s personal experiences, current breastfeeding duration was associated with the self-reported impact of the pandemic on medical counseling during breastfeeding. More specifically, 76% of the women who were breastfeeding for ≤6 weeks reported that the pandemic affected their medical counseling, as compared to 56% of the women breastfeeding between 6 weeks and 6 months and 18% of the women breastfeeding >6 months (*p* < 0.001). Likewise, women without breastfeeding experience were more likely to report that the pandemic affected their medical counseling during breastfeeding (49% vs. 38%; *p* < 0.001). In contrast, having a previous pregnancy experience was not associated with the self-reported impact of the pandemic on medical counseling during pregnancy (52% vs. 55%; *p* = 0.09).

### 3.6. Impact on Social Support during Lactation 

In total, 39% of the breastfeeding women reported having experienced the impact of the pandemic on the extent of social support they received during the breastfeeding period. Of those, women indicated to have received less support from family (87%), friends (87%), perinatal organizations (86%), and maternity assistance at home (68%) (see Table 3). Importantly, a substantial difference in the total number of women reporting to have experienced the impact of the pandemic on social support was observed according to the duration of breastfeeding (≤6 weeks: 73%; 6 w–6 m: 52%; >6 m: 12%; *p* < 0.001). Moreover, women without breastfeeding experience were more likely to report that the pandemic had an impact on social support during breastfeeding (45% vs. 34%; *p* < 0.001). 

## 4. Discussion

### 4.1. Main Findings 

The current study aimed to provide estimates of SARS-CoV-2 infections among pregnant and breastfeeding women, and to assess women’s perceived impact of the pandemic on their breastfeeding practices, medical counseling and social support during pregnancy and lactation. Therefore, an online survey was distributed through social media within the Belgian primary care setting a few weeks after the start of the lockdown (i.e., at the peak of the first wave of the pandemic). At that time, 0.3% of the more than 6400 pregnant and breastfeeding women reported to have tested positive for SARS-CoV-2. This percentage is substantially lower than the seroprevalence of 3–6% measured in the general population in Belgium in April 2020 [16]. Importantly, the estimates observed in our sample are undoubtedly underestimations of the actual prevalence due to the potential of an asymptomatic course of COVID-19 in pregnancy and to the lack of systematic testing in Belgium at the time of the survey completion [17,18]. However, even in the most extreme scenario when up to 85% of the infected pregnant respondents were asymptomatic [17,18], and were thus not tested and diagnosed, the seroprevalence of COVID-19 in our sample would not exceed the percentage observed in the general population [16]. In addition, the test positivity rate in our pregnant sample, despite the limited number of cases, is not higher than the rate obtained in the general population in the same period (i.e., 15–25%) [19]. Hence, our results do not indicate a higher susceptibility of pregnant women to contracting COVID-19. However, it cannot be ruled out that pregnant respondents imposed themselves additional restrictions during the lockdown to further minimize the risk of infection. 

Moreover, the small number of “confirmed” cases and the lack of outcome data prevented us from drawing conclusions on the severity of COVID-19 in pregnancy. Although some authors declared that pregnant women should not be considered a risk group for COVID-19 [20,21], other authors have argued that more evidence is required prior to ruling out an increased risk of adverse outcomes of COVID-19 in pregnancy [22,23]. Interestingly, and despite the subjectivity of the assessment and the limited number of cases, eight of the nine pregnant respondents with COVID-19 reported that their symptoms had a (rather) large influence on their functioning. This finding should at least remind us to not trivialize the potentially harmful impact of COVID-19 in pregnancy. Since the outbreak of the virus, several papers have been published on adverse outcomes of perinatal COVID-19, including miscarriage, fetal distress, preterm delivery, and maternal and neonatal death [3,11,24,25]. Larger cohorts and routine testing of all pregnant women are needed to correctly estimate the percentage of asymptomatic women and to calculate reliable prevalence and risk estimates [17,26,27]. 

In our cohort, we did not observe a negative impact of the lockdown on self-reported breastfeeding practices. Overall, more than 90% of the women refuted that the current situation affected the infant’s diet, nor indicated that the coronavirus was responsible for breastfeeding cessation. In fact, half of the women had (strongly) considered giving breastmilk for a longer period of time because of the virus, pointing towards positive breastfeeding perceptions triggered by the COVID-19 pandemic. Interestingly, house arrest during the lockdown could have had different effects on breastfeeding practices. Being more often at home clearly facilitated breastfeeding for some women, while others suffered from anxiety and stress due to concurrent childcare responsibilities. Hence, clinicians should be aware that the personal context and situation at home can affect women’s breastfeeding practices differently. 

Lastly, a substantial impact of the pandemic on medical counseling and support was observed, in line with a recent report from the UK [28]. Although the counseling of all types of HCPs was affected to some extent [29], the governmental perinatal organizations were most severely impacted by the measures taken. This is not surprising at all given that their consultation offices had to be closed for several weeks. Importantly, women without breastfeeding experience and in the early postpartum period experienced a higher burden in terms of reduced medical counseling and support. Although the latter finding is easy to understand given the higher number of consultations and support sessions early after birth, the results should encourage HCPs and perinatal organizations to specifically focus on both target groups in the future. 

### 4.2. Methodological Considerations 

Some strengths and limitations of the current study should be addressed. First, the study collected more than 6400 responses in a short study period of 8–10 days. Of those, more than 90% reported to not be involved in another COVID-19 research project, underlining that a large and unexplored perinatal population was included in our study to investigate COVID-19 epidemiology and self-reported consequences. As our study sample was collected in the primary care setting, it is likely that (severely) ill and hospitalized women did not complete the survey, and hence, were not included in the estimates of SARS-CoV-2 infections. However, the estimates observed in our sample would not be significantly changed if the number of hospitalized pregnant women currently registered in the Belgian COVID-19 obstetric surveillance system was considered [30]. 

Furthermore, limitations inherent to a self-reported questionnaire distributed via social media are applicable. Despite addressing a large and unexplored population living in all parts of Belgium, selection bias might have occurred. Compared to population data, respondents were slightly older, higher educated, and more professionally active, in particular in healthcare [31]. In addition, an overrepresentation of women breastfeeding for more than 6 months was noted [32]. The inclusion of this group of women with probably very positive feelings towards breastfeeding, along with the large number of women working in healthcare and survey distribution via breastfeeding fora, might have led to an underestimation of the impact of the COVID-19 pandemic on breastfeeding practices. However, given the vast majority of women reporting to have not experienced an adverse impact on their breastfeeding practices, it is plausible that our findings reflect the situation in the general breastfeeding population. In any case, the results were collected in a high-income country with a specific perinatal and child healthcare system. One should take into account that the results cannot simply be extended to other countries or settings. Finally, the survey did not include questions assessing the impact of the pandemic on social support during pregnancy; the study did also not provide comparisons with the non-COVID era with regard to women’s personal context or situation at home. 

### 4.3. Future Perspectives 

The current study aimed to promptly answer urgent questions of the public, HCPs, researchers, governmental perinatal organizations, and policymakers in Belgium with regard to the impact of the pandemic on breastfeeding practices, medical counseling and social support. Due to the study design, we were not able to collect data on breastfeeding initiation or duration among women who delivered or were breastfeeding while the containment and social isolation measures were in place. To assess the impact of these measures on breastfeeding initiation and duration, as compared to the non-COVID era, the annual breastfeeding statistics for Belgium are eagerly awaited. Future studies could also qualitatively investigate the breastfeeding intentions of mothers who delivered during the pandemic. Given the impact of the lockdown on women’s support and perinatal mental health [33,34], awareness of clinicians and governmental perinatal organizations is, however, required to ensure the emotional wellbeing of women and to safeguard perinatal and infant mental health in the wake of the pandemic. 

Overall, the results on the impact on medical counseling are informative for the Belgian context. However, given the global spread of SARS-CoV-2 and the worldwide imposed restrictions, it might be interesting to explore to what extent access to health services and social support has been affected across countries where different containment measures were in place.

Finally, it is likely that the organization of the healthcare system in Belgium will undergo sustainable transformations as a result of the pandemic, such as the expansion of telemedicine for non-urgent care visits (e.g., by midwives), and the integration of telemonitoring and mobile health applications in the follow-up of high-risk pregnancies [35,36,37]. It will be fascinating to monitor to what extent these virtual transformations will be adopted by the perinatal and child healthcare system and the public in Belgium. However, prior to nationwide implementation, a thorough investigation of the experiences, preferences and needs of HCPs and the public, as well as of the potential barriers and facilitators is required to ensure successful uptake across different disciplines and population groups.

## 5. Conclusions

In total, 0.3% of a cohort of pregnant and breastfeeding women residing in the primary care setting of Belgium tested positive for SARS-CoV-2 at the peak of the first wave of the pandemic. Although it cannot be ruled out that pregnant respondents imposed themselves additional restrictions to minimize the risk of infection, our results do not point towards a higher susceptibility of pregnant women to contracting COVID-19. Moreover, no negative impact of the lockdown on self-reported breastfeeding practices was observed. In fact, positive breastfeeding perceptions triggered by the coronavirus were cited by half of the women. In contrast, women’s medical counseling and social support were negatively affected by the lockdown, mainly among women without breastfeeding experience and in the early postpartum period. Hence, more consideration and alternative supportive manners such as tele-visits by midwives or perinatal organizations are required for these women in the wake of the pandemic and during subsequent waves. 

## Figures and Tables

**Table 1:** Estimates of SARS-CoV-2 infection in the study sample.

Estimated Outcomes		Pregnancy			Lactation	
	Total (*n* = 2602)	Dutch (*n* = 2303)	French (*n* = 299)	Total (*n* = 3817)	Dutch (*n* = 3284)	French (*n* = 533)
Respondents tested for SARS-CoV-2	2.7% (71)	2.5% (58)	4.3% (13)	3.7% (140)	3.3% (107)	6.2% (33)
Respondents tested positive for SARS-CoV-2	0.3% (9)	0.3% (7)	0.7% (2)	0.2% (8)	0.2% (5)	0.6% (3)
Test positivity rate in this sample	12.7% (9)	12.1% (7)	15.4% (2)	5.7% (8)	4.7% (5)	9.1% (3)
Hospitalized COVID-19 cases	11.1% (1)	0.0% (0)	50.0% (1)	0.0% (0)	0% (0)	0% (0)

Results are expressed as % (absolute numbers). “Dutch” and “French” refer to the language of the survey.

**Table 2:** Impact of the coronavirus pandemic on the extent of medical counseling during pregnancy and lactation.

Health Care Professional	More Follow-Up		Less Follow-Up		No Influence	
	Pregnancy	Lactation	Pregnancy	Lactation	Pregnancy	Lactation
Obstetrician	3.5% (47)	3.6% (42)	61.8 (819)	63.5% (746)	34.7% (460)	32.9% (387)
Midwife	7.6 (74)	7.7% (103)	64.5 (627)	73.0% (981)	27.9% (271)	19.3% (260)
General practitioner	8.2 (70)	5.1% (52)	42.1 (360)	53.4% (547)	49.8% (426)	41.5% (425)
Medical specialist	3.8 (20)	3.2% (23)	42.4 (226)	49.4% (351)	53.8% (287)	47.4% (337)
Pediatrician	N/A	4.8% (61)	N/A	54.0% (689)	N/A	41.2% (525)
Lactation consultant	N/A	6.9% (57)	N/A	59.3% (490)	N/A	33.8% (279)
Perinatal organization	N/A	3.9% (55)	N/A	84.3% (1195)	N/A	11.8% (168)

Results are expressed as % (absolute numbers). Percentages were calculated using as denominator the total number of women who reported to be counseled by a specific type of health care professional during pregnancy or breastfeeding. It is common practice in Belgium that pregnant and breastfeeding women have contact with different groups of health care professionals. N/A = not applicable.

**Table 3:** Impact of the coronavirus pandemic on the extent of social support during lactation.

Social Support Provided by	More Support	Less Support	No Influence
Family	6.1% (85)	87.2% (1214)	6.7% (93)
Friends	4.6% (62)	87.2% (1179)	8.2% (111)
Perinatal organization	2.9% (37)	86.1% (1092)	11.0% (140)
Maternity assistance at home	5.8% (51)	67.5% (597)	26.8% (237)

Results are expressed as % (absolute numbers). Percentages were calculated using as denominator the total number of women who indicated that a specific kind of support was applicable to them during the breastfeeding period.

## Supplementary Materials

The following are available online at https://www.mdpi.com/1660-4601/17/18/6766/s1, Table S1: Characteristics of the study participants; Table S2: Self-reported symptoms of the COVID-19 cases during pregnancy and lactation.

## References

[1] Zhu N., Zhang D., Wang W., Li X., Yang B., Song J., Zhao X., Huang B., Shi W., Lu R. (2020). A Novel Coronavirus from Patients with Pneumonia in China, 2019. N. Engl. J. Med..

[2] WHO WHO Director-General’s Opening Remarks at the Media Briefing on COVID-19—11 March 2020. https://www.who.int/dg/speeches/detail/who-director-general-s-opening-remarks-at-the-media-briefing-on-covid-19---11-march-2020.

[3] Chen H., Guo J., Wang C., Luo F., Yu X., Zhang W., Li J., Zhao D., Xu D., Gong Q. (2020). Clinical characteristics and intrauterine vertical transmission potential of COVID-19 infection in nine pregnant women: A retrospective review of medical records. Lancet.

[4] Zeng L., Xia S., Yuan W., Yan K., Xiao F., Shao J., Zhou W. (2020). Neonatal Early-Onset Infection with SARS-CoV-2 in 33 Neonates Born to Mothers With COVID-19 in Wuhan, China. JAMA Pediatr..

[5] Favre G., Pomar L., Musso D., Baud D. (2020). 2019-nCoV epidemic: What about pregnancies?. Lancet.

[6] Schwartz D.A., Graham A.L. (2020). Potential Maternal and Infant Outcomes from (Wuhan) Coronavirus 2019-nCoV Infecting Pregnant Women: Lessons from SARS, MERS, and Other Human Coronavirus Infections. Viruses.

[7] Wong S.F., Chow K.M., Leung T.N., Ng W.F., Ng T.K., Shek C.C., Ng P.C., Lam P.W., Ho L.C., To W.W. (2004). Pregnancy and perinatal outcomes of women with severe acute respiratory syndrome. Am. J. Obstet. Gynecol..

[8] Alfaraj S.H., Al-Tawfiq J.A., Memish Z.A. (2019). Middle East Respiratory Syndrome Coronavirus (MERS-CoV) infection during pregnancy: Report of two cases & review of the literature. J. Microbiol. Immunol. Infect..

[9] Liu H., Wang L.L., Zhao S.J., Kwak-Kim J., Mor G., Liao A.H. (2020). Why are pregnant women susceptible to COVID-19? An immunological viewpoint. J. Reprod. Immunol..

[10] Yu N., Li W., Kang Q., Xiong Z., Wang S., Lin X., Liu Y., Xiao J., Liu H., Deng D. (2020). Clinical features and obstetric and neonatal outcomes of pregnant patients with COVID-19 in Wuhan, China: A retrospective, single-centre, descriptive study. Lancet Infect. Dis..

[11] Baud D., Greub G., Favre G., Gengler C., Jaton K., Dubruc E., Pomar L. (2020). Second-Trimester Miscarriage in a Pregnant Woman With SARS-CoV-2 Infection. JAMA.

[12] Yang H., Hu B., Zhan S., Yang L.-Y., Xiong G. (2020). Effects of SARS-CoV-2 infection on pregnant women and their infants: A retrospective study in Wuhan, China. Arch. Pathol. Lab. Med..

[13] Schoenmakers S., Snijder P., Verdijk R., Kuiken T., Kamphuis S., Koopman L., Krasemann T., Rousian M., Broekhuizen M., Steegers E. (2020). SARS-CoV-2 placental infection and inflammation leading to fetal distress and neonatal multi-organ failure in an asymptomatic woman. medRxiv.

[14] World Health Organization Media Statement: Knowing the Risk for COVID-19. https://www.who.int/indonesia/news/detail/08-03-2020-knowing-the-risk-for-covid-19.

[15] Siedner M.J., Kraemer J.D., Meyer M.J., Harling G., Mngomezulu T., Gabela P., Dlamini S., Gareta D., Majozi N., Ngwenya N. (2020). Access to primary healthcare during lockdown measures for COVID-19 in rural South Africa: A longitudinal cohort study. medRxiv.

[16] Herzog S., De Bie J., Abrams S., Wouters I., Ekinci E., Patteet L., Coppens A., De Spiegeleer S., Beutels P., Van Damme P. (2020). Seroprevalence of IgG antibodies against SARS coronavirus 2 in Belgium: A prospective cross-sectional nationwide study of residual samples. medRxiv.

[17] Khalil A., Hill R., Ladhani S., Pattisson K., O’Brien P. (2020). Severe acute respiratory syndrome coronavirus 2 in pregnancy: Symptomatic pregnant women are only the tip of the iceberg. Am. J. Obstet. Gynecol..

[18] Sutton D., Fuchs K., D’Alton M., Goffman D. (2020). Universal Screening for SARS-CoV-2 in Women Admitted for Delivery. N. Engl. J. Med..

[19] Sciensano.be/sites/default/files/Covid19/COVID-19_Weekly%20report_20200423%20-%20NL_0.pdf.

[20] Teles Abrao Trad A., Ibirogba E.R., Elrefaei A., Narang K., Tonni G., Picone O., Suy A., Carreras Moratonas E., Kilby M.D., Ruano R. (2020). Complications and outcomes of SARS-CoV-2 in pregnancy: Where and what is the evidence?. Hypertens. Pregnancy.

[21] Trocado V., Silvestre-Machado J., Azevedo L., Miranda A., Nogueira-Silva C. (2020). Pregnancy and COVID-19: A systematic review of maternal, obstetric and neonatal outcomes. J. Matern. Fetal Neonatal Med..

[22] Collin J., Byström E., Carnahan A., Ahrne M. (2020). Public Health Agency of Sweden’s Brief Report: Pregnant and postpartum women with severe acute respiratory syndrome coronavirus 2 infection in intensive care in Sweden. Acta Obstet. Gynecol. Scand..

[23] Barbero P., Mugüerza L., Herraiz I., García Burguillo A., San Juan R., Forcén L., Mejía I., Batllori E., Montañez M.D., Vallejo P. (2020). SARS-CoV-2 in pregnancy: Characteristics and outcomes of hospitalized and non-hospitalized women due to COVID-19. J. Matern. Fetal Neonatal Med..

[24] Hantoushzadeh S., Shamshirsaz A.A., Aleyasin A., Seferovic M.D., Aski S.K., Arian S.E., Pooransari P., Ghotbizadeh F., Aalipour S., Soleimani Z. (2020). Maternal death due to COVID-19. Am. J. Obstet. Gynecol..

[25] Zaigham M., Andersson O. (2020). Maternal and perinatal outcomes with COVID-19: A systematic review of 108 pregnancies. Acta Obstet. Gynecol. Scand..

[26] Lopes de Sousa Á.F., Carvalho H.E.F., Oliveira L.B., Schneider G., Camargo E.L.S., Watanabe E., de Andrade D., Fernandes A.F.C., Mendes I.A.C., Fronteira I. (2020). Effects of COVID-19 Infection during Pregnancy and Neonatal Prognosis: What Is the Evidence?. Int. J. Environ. Res. Public Health.

[27] Panchaud A., Favre G., Pomar L., Vouga M., Aebi-Popp K., Baud D. (2020). An international registry for emergent pathogens and pregnancy. Lancet.

[28] Vazquez-Vazquez A., Dib S., Rougeaux E., Wells J.C., Fewtrell M. (2020). The impact of the Covid-19 lockdown on the experiences and feeding practices of new mothers in the UK: Preliminary data from the COVID-19 New Mum Study. medRxiv.

[29] Verhoeven V., Tsakitzidis G., Philips H., Van Royen P. (2020). Impact of the COVID-19 pandemic on the core functions of primary care: Will the cure be worse than the disease? A qualitative interview study in Flemish GPs. BMJ Open.

[30] Belgian Obstetric Surveillance System Dashboard COVID-19. https://www.b-oss.be/covid-results.

[31] Studiecentrum Voor Perinatale Epidemiologie Perinatale Activiteiten in Vlaanderen 2018. https://www.zorg-en-gezondheid.be/sites/default/files/atoms/files/EMBARGO_SPE_Perinatale%20activiteiten%20in%20Vlaanderen%202018.pdf.

[32] Kind en Gezin Cijfers en Rapport: Kind in Vlaanderen 2018. https://www.kindengezin.be/img/KIN0529_KIV2018_Alles.pdf.

[33] Ceulemans M., Hompes T., Foulon V. (2020). Mental health status of pregnant and breastfeeding women during the COVID-19 pandemic: A call for action. Int. J. Gynaecol. Obstet..

[34] Davenport M.H., Meyer S., Meah V.L., Strynadka M.C., Khurana R. (2020). Moms Are Not OK: COVID-19 and Maternal Mental Health. Front. Glob. Womens Health.

[35] Mann D.M., Chen J., Chunara R., Testa P.A., Nov O. (2020). COVID-19 transforms health care through telemedicine: Evidence from the field. J. Am. Med. Inform. Assoc..

[36] Khalil A., Perry H., Lanssens D., Gyselaers W. (2019). Telemonitoring for hypertensive disease in pregnancy. Expert Rev. Med. Devices..

[37] Gyselaers W., Lanssens D., Perry H., Khalil A. (2019). Mobile Health Applications for Prenatal Assessment and Monitoring. Curr. Pharm. Des..

