# Supplementary material for: SARS-CoV-2 Infections and Impact of the COVID-19 Pandemic in Pregnancy and Breastfeeding: Results from an Observational Study in Primary Care in Belgium
Source: Int J Environ Res Public Health. 2020 Sep 17;17(18):6766. doi: 10.3390/ijerph17186766 (PMC7559009; doi:10.3390/ijerph17186766)
Supplement: Supplementary file 1 [file ijerph-17-06766-s001.pdf]

Table S1. Characteristics of the study participants.

Characteristics	PREGNANT WOMEN			BREASTFEEDING WOMEN		
	**Total (n=2647)**	**Dutch (n=2344)**	**French (n=303)**	**Total (n=3823)**	**Dutch (n= 3290)**	**French (n=533)**
Age						
<20	0.0% (0)	0.0% (0)	0.0% (0)	<0.1% (1)	<0.1% (1)	0.0% (0)
20-24	2.3% (61)	2.4% (55)	2.0% (6)	1.4% (53)	1.4% (45)	1.5% (8)
25-29	29.7% (782)	29.9% (699)	27.7% (83)	21.6% (823)	21.5% (707)	21.9% (116)
30-34	52.4% (1381)	52.8% (1235)	48.7% (146)	52.3% (1994)	53.1% (1743)	47.4% (251)
35 or older	15.7% (413)	14.9% (348)	21.7% (65)	24.7% (941)	23.9% (786)	29.2% (155)
Highest degree						
Higher education	84.4% (2226)	84.5% (1980)	83.7% (246)	81.5% (3110)	81.4% (2679)	81.9% (431)
No higher education	15.6% (412)	15.5% (364)	16.3% (48)	18.5% (706)	18.6% (611)	18.1% (95)
Occupation						
Professionally active	94.1% (2478)	94.9% (2213)	87.5% (265)	90.9% (3438)	92.2% (3001)	82.8% (437)
Not professionally active	5.9% (156)	5.1% (118)	12.5% (38)	9.1% (346)	7.8% (255)	17.2% (91)
Marital status						
Partner	96.9% (2564)	97.6% (2287)	91.4% (277)	96.4% (3684)	97.1% (3194)	91.9% (490)
No partner	3.1% (83)	2.4% (57)	8.6% (26)	3.6% (137)	2.9% (94)	8.1% (43)
Gestational trimester						
Trimester 1 (0-12w)	12.2% (321)	12.6% (293)	9.2% (28)	N/A	N/A	N/A
Trimester 2 (13-27w)	42.8% (1124)	42.8% (994)	42.9% (130)	N/A	N/A	N/A
Trimester 3 (28-40w)	45.0% (1180)	44.6% (1035)	47.9% (145)	N/A	N/A	N/A
Gravidity						
Primigravida	48.6% (1278)	49.1% (1141)	45.2% (137)	N/A	N/A	N/A
Multigravida	51.4% (1349)	50.9% (1183)	54.8% (166)	N/A	N/A	N/A
Planned pregnancy						
Yes	92.9% (2440)	94.0% (2185)	84.2% (255)	N/A	N/A	N/A
No	7.1% (187)	6.0% (139)	15.8% (48)	N/A	N/A	N/A
Pregnancy follow-up mainly by						
Obstetrician	86.3% (2249)	86.3% (1989)	85.8% (260)	N/A	N/A	N/A
Midwife	10.9% (284)	10.5% (242)	13.9% (42)	N/A	N/A	N/A
General practitioner	2.4% (63)	2.7% (62)	0.3% (1)	N/A	N/A	N/A
Other	0.4% (11)	0.5% (11)	0% (0)	N/A	N/A	N/A
Previous breastfeeding experience						
Yes	N/A	N/A	N/A	54.5% (2011)	54.7% (1739)	53.5% (272)
No	N/A	N/A	N/A	45.5% (1679)	45.3% (1443)	46.5% (236)

Results are expressed as % (absolute numbers). N/A = not applicable

Table S2. Self-reported symptoms of the COVID-19 cases during pregnancy and lactation.

Symptoms	TOTAL	PREGNANCY			LACTATION		
	(n=17)	Total (n=9)	Dutch (n=7)	French (n=2)	Total (n=8)	Dutch (n= 5)	French (n=3)
Headache	**13 (76.5%)**	7 (77.8%)	6 (85.7%)	1 (50.0%)	6 (75.0%)	3 (60.0%)	3 (100.0%)
Fever	**12 (70.6%)**	7 (77.8%)	5 (71.4%)	2 (100.0%)	5 (62.5%)	3 (60.0%)	2 (66.7%)
Anosmia/dysgeusia/anorexia	**11 (64.7%)**	5 (55.6%)	5 (71.4%)	0 (0.0%)	6 (75.0%)	3 (60.0%)	3 (100.0%)
Muscle pain	**11 (64.7%)**	6 (66.7%)	5 (71.4%)	1 (50.0%)	5 (62.5%)	3 (60.0%)	2 (66.7%)
Cough	**9 (52.9%)**	6 (66.7%)	5 (71.4%)	1 (50.0%)	3 (37.5%)	2 (40.0%)	1 (33.3%)
Runny nose	**9 (52.9%)**	6 (66.7%)	6 (85.7%)	0 (0.0%)	3 (37.5%)	2 (40.0%)	1 (33.3%)
Shortness of breath	**8 (47.1%)**	5 (55.6%)	4 (57.1%)	1 (50.0%)	3 (37.5%)	2 (40.0%)	1 (33.3%)
Sore throat	**6 (35.3%)**	5 (55.6%)	4 (57.1%)	1 (50.0%)	1 (12.5%)	1 (20.0%)	0 (0.0%)
Watery diarrhea	**4 (23.5%)**	3 (33.3%)	2 (28.6%)	1 (50.0%)	1 (12.5%)	1 (20.0%)	0 (0.0%)

Results are expressed as absolute numbers (%).
